# Editorial: Large-scale recording of neuronal activity at high spatiotemporal resolutions and applications in neuroscience

**DOI:** 10.3389/fnins.2023.1202207

**Published:** 2023-05-10

**Authors:** Bo Li, Lingjie Kong, Kiryl D. Piatkevich, Hod Dana

**Affiliations:** ^1^State Key Laboratory of Medical Neurobiology, Department of Neurology, Ministry of Education (MOE), Frontiers Center for Brain Science, Institute for Translational Brain Research, Huashan Hospital, Fudan University, Shanghai, China; ^2^State Key Laboratory of Precision Measurement Technology and Instruments, Department of Precision Instrument, Tsinghua University, Beijing, China; ^3^IDG/McGovern Institute for Brain Research, Tsinghua University, Beijing, China; ^4^School of Life Science, Westlake University, Hangzhou, Zhejiang, China; ^5^Westlake Laboratory of Life Sciences and Biomedicine, Hangzhou, Zhejiang, China; ^6^Institute of Basic Medical Sciences, Westlake Institute for Advance Study, Hangzhou, Zhejiang, China; ^7^Department of Neurosciences, Lerner Research Institute, Cleveland Clinic, Cleveland, OH, United States; ^8^Department of Molecular Medicine, School of Medicine, Cleveland Clinic Lerner College of Medicine, Case Western Reserve University, Cleveland, OH, United States

**Keywords:** non-linear microscopy techniques, two-photon microscopy (TPM), light-sheet microscopy (LSM), wide-field microscopy (WFM), light-field microscopy, large scale microscopy

Accurate detection of brain-wide interactions across local neural circuits can revolutionize our understanding of dynamic signaling within the nervous system. Developing such capabilities necessitates the development and implementation of novel technologies and approaches. Although optical methods may satisfy the fundamental prerequisites for achieving high spatiotemporal resolution, currently available imaging techniques are constrained by inherent limitations arising from tissue properties and imaging hardware. Consequently, existing imaging systems must optimize their scale, resolution, speed, and depth for monitoring the whole, or a substantial part, of the brain of large model organisms. Recent advancements toward these goals include the development of new genetically encoded neuronal activity indicators and the proposal of complementary optical imaging methods. Techniques such as light sheet microscopy, light field microscopy, and wide field microscopy enable whole-brain or whole-animal recordings with single neuron resolution in small model organisms like *C. elegans* and zebrafish. *In vivo* imaging of neural activity in behaving mice is also possible through miniscopes and multiphoton microscopy. The incorporation of advanced computational imaging techniques can further enhance imaging performance via more efficient acquisition schemes. To simultaneously capture cellular-level activity patterns across large portions of the mammalian brain, additional innovative approaches are necessary.

The articles featured in this Research Topic provide a focused and updated overview of current methods and results in neuronal activity recording. Wang et al. introduce naturally modulated inverted light-sheet microscopy (NM-ILSM), a technique that improves axial resolution by 15% while maintaining a wide field-of-view (FOV) and enhancing imaging contrast 5 fold through background suppression. This technology enables convenient imaging quality improvement for uncleared tissue and expands the biological application scope of ILSM (Wang et al.). Zhai et al. present structured-illumination and interleaved-reconstruction based Fourier light field microscopy (SI-FLFM), which suppresses background fluorescence, leading to tens of times improved signal-to-background ratios without sacrificing imaging speed. This technology is suitable for applications requiring weak fluorescence signals and high imaging speed (Zhai et al.). Ding et al. develop a multicolor wide-field large-volume tomography (multicolor WVT) to simultaneously acquire fluorescent signals in blue, green, and red channels in the whole brain, demonstrating the system's potential in deciphering between multiple neural circuit components (Ding et al.). Das et al. compare two novel calcium-modulated photoactivatable ratiometric integrators (CaMPARI) and find a surprising conclusion: CaMPARI2, the latest version of CaMPARI, exhibits lower photoconversion efficiency in active neurons in the mouse cortex and hippocampus than CaMPARI1. They argue that some sensor characteristics were not accurately predicted by *in vitro* screening assays during CaMPARI2's optimization process, emphasizing the need for *in vivo* screening and validation in future optimization attempts to enhance screening pipeline predictability (Das et al.). Chen et al. review current optical miniscopes for *in vivo* neural activity imaging in freely moving animals, discussing both single-photon and multiphoton excitation strategies, fundamental principles, system structures, and technical advancements. These optical miniscopes are becoming lighter, more colorful, offering larger FOVs, better signal-to-noise ratios, and deeper imaging, making them increasingly suitable for *in vivo* neural activity imaging in freely moving animals (Chen et al.). Lastly, Xiao et al. review three-photon excited fluorescence imaging as a novel tool for deep *in vivo* imaging. As adaptive excitation, adaptive optics, and other strategies are proposed to enhance performance, three-photon excited fluorescence imaging has achieved remarkable imaging depths in various animal models (including mice, rats, Drosophila, and zebrafish) and brain regions (such as the hippocampus and spinal cord). It is highly plausible that additional optical tools and approaches will be developed for large-scale neuronal activity recording at high spatiotemporal resolutions and applications in neuroscience (Xiao et al.).

In conclusion, this Research Topic provides an updated overview of imaging methods optimized for neuronal activity recording, showcasing some of the latest improvements in light-sheet microscopy, structured-illumination microscopy, multicolor wide-field large-volume tomography, miniscopes, and three-photon fluorescence imaging. In perspective, we believe that these technological advancements represent an important step toward development of advanced imaging techniques for large-scale neuronal activity recording at high spatiotemporal resolutions for neuroscience research.

## Author contributions

BL wrote the first draft of this editorial and all authors provided their comments. All authors contributed to the article and approved the submitted version.

